# Ashwagandha-Induced Programmed Cell Death in the Treatment of Breast Cancer

**DOI:** 10.3390/cimb46070454

**Published:** 2024-07-18

**Authors:** Renata Kołodziejska, Agnieszka Tafelska-Kaczmarek, Mateusz Pawluk, Krzysztof Sergot, Lucyna Pisarska, Alina Woźniak, Hanna Pawluk

**Affiliations:** 1 Department of Medical Biology and Biochemistry, Faculty of Medicine, Collegium Medicum in Bydgoszcz, Nicolaus Copernicus University in Toruń, Karłowicza 24, 85-092 Bydgoszcz, Poland; pawluk.mateusz23@gmail.com (M.P.); lucyna.pisarska@cm.umk.pl (L.P.); hannapawluk1@wp.pl (H.P.); 2 Department of Organic Chemistry, Faculty of Chemistry, Nicolaus Copernicus University, Gagarina 7, 87-100 Toruń, Poland; tafel@umk.pl; 3 Laboratory of Laser Molecular Spectroscopy, Institute of Applied Radiation Chemistry, Faculty of Chemistry, Lodz University of Technology, Wroblewskiego 15, 93-590 Lodz, Poland; krzysztofsergot@gmail.com

**Keywords:** *Withania somnifera*, Withaferin A, breast cancer, apoptosis, cell death

## Abstract

The aim of this review is to provide experimental evidence for the programmed-death activity of Ashwagandha (*Withania somnifera*) in the anti-cancer therapy of breast cancer. The literature search was conducted using online electronic databases (Google Scholar, PubMed, Scopus). Collection schedule data for the review article covered the years 2004–2024. Ashwagandha active substances, especially Withaferin A (WA), are the most promising anti-cancer compounds. WS exerts its effect on breast cancer cells by inducing programmed cell death, especially apoptosis, at the molecular level. Ashwagandha has been found to possess a potential for treating breast cancer, especially estrogen receptor/progesterone receptor (ER/PR)-positive and triple-negative breast cancer.

## 1. Introduction

Cancer affects both women and men and constitutes a serious clinical problem, as cancer incidence and mortality rates are increasing every year. According to the International Agency for Research on Cancer (IARC), almost 20 million new cancer cases and 9.7 million cancer-related deaths were reported in 2022. Estimates show that approximately 1 in 9 men and 1 in 12 women die from cancer. In 2022, the most frequently diagnosed cancer was lung cancer, corresponding to 12.4% of all cancers in the world, followed by breast cancer in women (11.6%), colorectal cancer (9.6%), prostate cancer (7.3%), and stomach (4.9%) [1].

The 2024 Cancer Statistics Update from the American Cancer Society estimates that 2,001,140 new cases of cancer and 611,720 cancer deaths are projected to occur in the United States. Incidence rates from 2015 to 2019 increased by 0.6–1% per year for breast, pancreatic, and endometrial cancers and by 2–3% per year for papillomavirus-related prostate, liver, kidney, and oral cancers and melanoma [2,3].

The main causes of cancer, apart from genetics, may be increasing environmental pollution, improper diet, low physical activity, alcohol abuse or smoking. Exposure of ancestors to toxic substances, stress, or improper nutrition may influence the occurrence of epigenetic intergenerational inheritance of cancer and the emergence of a variable patient phenotype. Breast cancer (BC) is the most common malignant tumor and the main cause of death in women, and pathogenic variants in BC susceptibility genes constitute the strongest hereditary risk factor for the development of the disease, especially in the context of early-onset breast cancer (EOBC), which is inherited in approximately 10–20% [4,5]. BC is a heterogeneous group of tumors that differ in their morphological appearance, clinical course, and prognosis. The following molecular subtypes of breast cancer are distinguished: luminal A (60–70%), luminal B (10–20%), human epidermal growth factor (HER2) positive (13–15%), and triple-negative breast cancer (TNBC; 10–15%). In routine practice, molecular forms of breast cancer are defined on the basis of immunohistochemical examination, assessing the expression of the following proteins: estrogen receptor (ER), progesterone receptor (PR), and HER2 receptor [6,7].

There is a great need to develop effective and easily accessible methods for early detection of this disease. The mammography examination (MMG) used so far is not sensitive enough to detect all cases of breast cancer, although it is still a standard examination and the most frequently used in diagnostics. In addition to MMG, other imaging methods are used to diagnose breast cancer, ultrasound (USG), magnetic resonance imaging (MRI), and positron emission tomography (PET) [8,9,10].

The treatment system is based on clinical and pathological assessment, taking into account the histological type and degree of cancer malignancy, biomarker expression, advancement of the primary tumor location, and the extent of metastases. Apart from invasive treatment and chemotherapy, pharmacological treatment is used. The development of personalized medicine and immunotherapy in recent years has resulted in significant progress in breast cancer treatment [11].

Apart from conventional medicine, natural medicine is becoming more and more popular due to its lower toxicity and selectivity compared to conventional therapies. One of the medicinal plants used in traditional medicine is *Withania somnifera* (WS) [12]. Ashwagandha is mostly considered safe, but side effects are possible [13].

Preclinical experimental evidence shows that WS leaf and root extracts inhibit cancer [14,15,16,17,18,19]. Methanol and ethanol extracts of WS stems have been shown to be highly cytotoxic and inhibit the growth of the human breast cancer cell line [20]. Experimental studies on animals have proven that the use of WS root extract and intermittent fasting are a promising solution in the treatment of breast cancer to overcome cisplatin resistance [21]. In turn, a standardized Ashwagandha extract (Oncowithanib) showed effective therapeutic activity in MCF7 cells and is associated with suppression of the expression of key cellular kinases, such as RSK1, AKT1, and mTOR [22].

It is known that WS has anticancer properties, but the mechanism of its action is not fully understood. The inhibition of cancer cells by WA may be the result of the following molecular mechanisms: induction of apoptosis; induction of oxidative stress; reduction of NF-κB, STAT3, and estrogen receptor expression; inhibition of the cell cycle in the G2-M phase, proteasomes, and processes important for the spread of cancer cell metastases [16,23,24,25,26,27,28,29,30,31,32,33,34,35,36,37,38,39,40,41,42,43,44,45,46,47,48,49,50,51]. WA inhibits cancer metastasis by partially reversing the epithelial-to-mesenchymal pathway, targeting the urokinase-type plasminogen activator (uPA) and activating the transcription factors Notch2 and Notch4 and reducing Notch1 [39,41,43].

This review assessed the anti-tumor role of WS in breast cancer based on the available scientific literature. We tried to determine the involvement of one of the most active components of Ahwagandha, withaferin A (WA), in the induction of programmed cell death, one of the mechanisms of inhibiting cancer cell proliferation and metastasis.

## 2. Data Collection Methodology

This review is narrative in nature and presents the collected literature on Ashwagan-dha-induced programmed tumor-cell death in breast cancer. A comprehensive search strategy was employed across international databases like Google Scholar, PubMed, and Scopus using keywords and phrases, namely *Withania somnifera* L. Dunal in breast cancer; Withaferin A, Ashwagandha, Ashwaganddha in breast cancer; apoptosis by Ashwagandha in breast cancer; and programmatic death of breast cancer. The collection schedule data for the review article covered the years 2004–2024, with a total of 242 articles. The publication inclusion criteria were as follows: (1) articles on Ashwagandha in the treatment of breast cancer, with particular emphasis on the mechanism of promoted cancer cell death; (2) only article and review document types (preclinical and clinical research) and; and (3) only English-language articles. The exclusion criteria were as follows: (1) articles that did not concern the use of Ashwagandha in the treatment of breast cancer; (2) articles that have not been peer-reviewed.

## 3. *Withania somnifera*

Ashwagandha (*Withania somnifera* L. *Dunal*) is a species of plant from the Solanaceae family. It naturally occurs in dry regions of tropical and subtropical climates, i.e., in Africa, Asia, and southern Europe. The name “Ashwagandha” was derived from the Sanskrit terms “ashva”, which means “horse,” and “gandha”, which means “smell”, and denotes the root’s aroma, which is similar to that of a horse. Synonyms of the name Ashwagandha are Indian winter cherry, Indian ginseng, and poison gooseberry. The plant has derived its names due to its certain features. Because of its pronounced anti-stress effects, the plant was ascribed its species name “somnifera”, meaning “sleep-inducer” in Latin [52]. The roots, leaves, flowers, and fruits of this plant are used in Indian folk medicine (Ayurveda) and as a dietary supplement [53,54].

WS and its roots have a wide range of pharmacological effects due to their medically useful chemical composition. It is active, among others, with the following properties: anti-inflammatory, anti-arthritic, anti-cancer, anti-epileptic, anti-depressant, anti-Alzheimer’s disease, anti-Parkinson’s disease, analgesic, cardioprotective, neuroprotective, anti-microbial, anti-fungal, anti-oxidant, anti-diabetic agent, etc. [44,55].

Various chemical components are found in the different parts of the plant. The roots of WS contain alkaloids (withananine, withanine, somniferine, somnine, somniferinine), amino acids (alanine, cysteine, glycine, tyrosine), steroidal lactones (withaferin A, withanolides A, B, D, E, F, G, H, I, J, K, L, M), steroids (sitoindosides VII, VIII, IX, X), volatile oil, starch, reducing sugars, glycosides, hentriacontane, dulcitol, flavonoids, withaniol, withanicil, iron, withasomnine, withanosides I-VII, and many others [52,56,57,58,59]. Typically, WS roots are used for medicinal purposes in the form of root extract as well as powder. Ashwagandha leaves contain withaferin A; 12 withanolides, including withanolide A, B, D, and E; withanone, flavonoids; free amino acids; and also condensed tannins, chlorogenic acid, *N*-heterocyclic compounds, and others [52,57,58]. Fruits, flowers, and seeds contain compounds like withanolides, withanolide glycosides, withanone, amino acids, flavonoids, condensed tannins, proteolytic eznyme, and psoralen [52,57,58,60].

**Figure 1:**
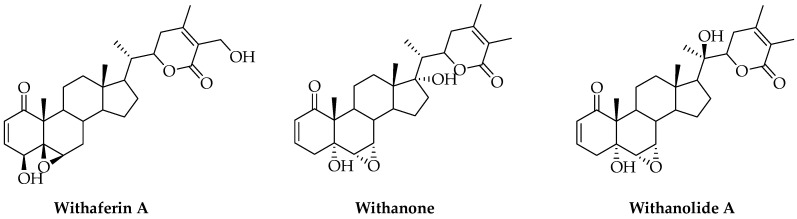
Structures of key components present in *Withania somnifera*.

The major chemical constituents of Ashwagandha are withanolides, C28-steroidal lactones, having an ergostane skeleton, in which C-22 and C-26 are appropriately oxidized to form five- or six-membered lactone rings [57]. Moreover, various compounds were found in WS, which are the modifications or structural variants of withanolides such as withaferin A, withanone, ashwagandhanolide, and sitoindosides. The main components of Withania somnifera are shown in Figure 1.

*Withania somnifera* extract has anti-oxidant and anti-microbial properties [61]. It has been found that WS shows activity against a variety of bacteria, viruses, and fungi, mainly due to the presence of withaferin A, withantolide A, and withanone [62,63,64]. Moreover, anti-inflammatory activity against protein denaturation in vitro results from Ashwagandha’s alkaloids and withanolides [65]. Many studies have also confirmed that the main components of Ashwagandha are responsible for the neuroprotective and anti-neurodegenerative effects in diseases such as Alzheimer’s, Parkinson’s, Huntington’s, and epilepsy [66,67,68,69,70,71,72]. Withaferin A from WS has been shown to help treat diabetes [73,74]. In turn, the alkaloids present in the WS plant have anti-stress activity [75,76]. Extensive research is also being conducted on the anti-cancer properties of Ashwagandha extract, especially its phytochemicals withanolides and withaferin A [17]. Activity has been found against cancer of prostate, kidney, bladder, gastric, colon, lung, breast, leukemia, and others [12,77,78].

## 4. Mechanism of Breast Cancer Cell Death by Ashwagandha

WS is involved in many biochemical processes, including apoptosis, which is a form of cell suicide that occurs not only as a result of cell damage or external stress but also during normal development and morphogenesis.

**Figure 2:**
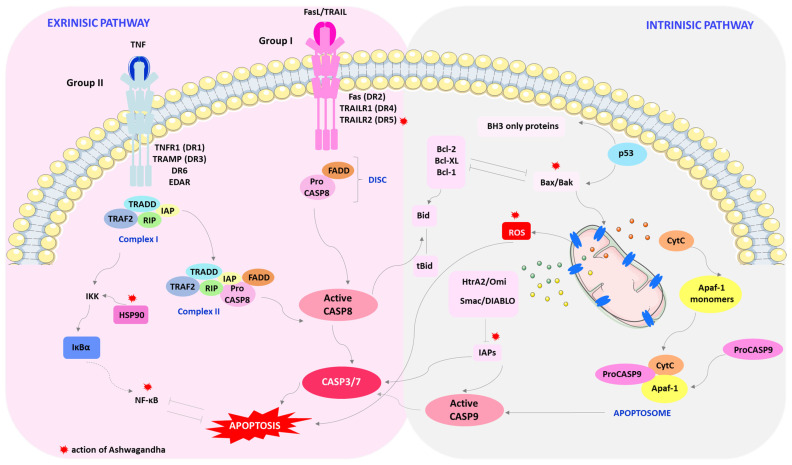
Internal and external pathways of apoptosis: the action of Ashwagandha. The effects of Ashwagandha on pathways of apoptosis are marked with a red asterisk. Active components of Ashwagandha induce apoptosis in both the internal and external pathways by mediating the production of reactive oxygen species and regulating the expression of Bcl-2 and IAP family proteins and the heat shock protein HSP90, as well as activating Death Receptor 5 (DR5) and inhibiting the IKK/NF-κB pathway. Abbreviations: Apaf-1—apoptotic protease activating factor 1; Bak, Bax, Bcl-1, Bcl-XL, Bid, tBid—Bcl-2 family proteins; BH3-only proteins—Bcl-2 homology 3; CASP3/7/8/9—caspase-3/7/8/9; CytC—cytochrome C; FADD—Fas-associated death domain; Fas-L—Fas ligand; IAPs—inhibitors of apoptosis proteins; HSP90—heat shock protein 90; HtrA2/Omi—apoptosis proteins; IκBα—nuclear factor of kappa light polypeptide gene enhancer in B-cells inhibitor alpha; IKK—kinase complex; NF-κB—nuclear factor-κB; ProCASP9—procaspase-9; Fas (DR2) TRAILR1 (DR4), TRAILR2 (DR5), TNFR1 (DR1), TRAMP (DR3), DR6 and EDAR—tumor necrosis factor receptor TNFR family; RIP—receptor-interacting protein kinase; ROS—reactive oxygen species; Smac/DIABLO—apoptosis proteins; TRAF2—TNF receptor associated factor-2; TRAIL—TNF-related apoptosis-inducing ligand; TNF—tumor necrosis factor; TRADD—TNF-related death domain. This figure was created using Servier Medical Art (available at https://smart.servier.com/, accessed on 12 April 2024).

The mechanism of apoptosis mainly consists of two basic pathways involved in inducing apoptosis, intracellular (intrinsic apoptosis) or extracellular (extrinsic apoptosis) (Figure 2) [79].

In the intrinsic pathway, apoptosis occurs via mitochondrial mediation. Apoptotic signaling in the extrinsic pathway involves extracellular ligands such as tumor necrosis factor (TNF), Fas ligand (Fas-L), and TNF-related apoptosis-inducing ligand (TRAIL). These ligands are attached to the extracellular domain of transmembrane receptors (DRs), which include TNF type 1 receptor (TNFR1), Fas (also called CD95/Apo-1), and TRAIL receptors [80].

The intrinsic, mitochondrial pathway of apoptosis is triggered by various intracellular stressors. Proteins from the Bcl-2 family are responsible for regulating this pathway, including pro-apoptotic proteins (Bax, Bak, Bok, Bim, Bid, Bik, Bad, Bmf, Hrk, Noxa, Puma, Blk) and anti-apoptotic proteins (Bcl-2, Bcl-XL, Bcl-w, A1, Mcl-1). Internal cellular stress activates Bcl-2 proteins that have only one domain, the so-called BH3-only proteins (Bcl-2 homology 3), containing Bid, Bim, Bad, Bik, Noxa, Puma, and Hrk. These proteins neutralize the action of anti-apoptotic proteins or, acting directly, activate multi-domain apoptosis-inducing proteins, i.e., Bax or Bak [81,82,83,84,85].

Oligomerizing Bax or Bak create pores in the outer mitochondrial membrane, which results in its destabilization. Disruption of the integrity of mitochondrial potential results in the release of pro-apoptotic proteins into the cytosol. Proteins that are generally involved in intrinsic pathway include cytochrome C (called apoptotic protease-activating factor 1 (Apaf-2)), Smac/DIABLO, HtrA2/Omi and AIF, Endonuclease G and CAD. Cytochrome C (CytC) combines with Apaf-1 and procaspase-9 to form an apoptosome that releases caspase-9 (CASP9). In turn, the CASP9 initiator activates caspase-3 (CASP3) and caspase-7 (CASP7). Smac/DIABLO and HtrA2/Omi indirectly promote apoptosis by affecting caspase-3/7 activation through inhibition of inhibitor of apoptosis proteins (IAPs). Whereas, AIF, Endonuclease G, and CAD proteins travel directly to the cell nucleus, cutting DNA into short fragments [86].

In the intrinsic apoptosis pathway, the element of pro-apoptotic regulation is the p53 protein, the so-called guardian of the genome. Under stress conditions, the p53 protein moves to the mitochondria, where it forms a complex with Bcl-2 and Bcl-XL, causing their inactivation. As a result, the permeability of the mitochondrial membrane increases and CytC is released [87].

In the extrinsic apoptosis pathway, the pro-apoptotic signal is transmitted via receptors from the tumor necrosis factor receptor TNFR family (Fas (DR2) TRAILR1 (DR4), TRAILR2 (DR5), TNFR1 (DR1), TRAMP (DR3), DR6 and EDAR). A feature of death receptors is the presence of an intracellular domain called the death domain (DD). After attaching the ligand to the receptor, it is activated through oligomerization and conformational changes, which then attaches an adapter protein such as Fas-associated death domain (FADD), tumor necrosis factor receptor-1-associated death domain (TRADD), or death domain-associated protein (Daxx)-containing death domains. The adapter protein further combines with procaspase-8 to form the death-inducing signaling complex (DISC). Attachment of an appropriate ligand to the receptor induces signal transmission through the death domain of the adapter protein to caspase-8 (CASP8). The consequence of this is the activation by autoproteolysis of procaspase-8, which is a direct activator of caspase 3/7 [80,81,84,88].

Fas (DR2), TRAILR1 (DR4), and TRAILR2 (DR5) receptors belong to the first group of death receptors activated by FasL and TRAIL. Once activated, these receptors recruit the DISC complex, which consists of FADD and CASP8. Due to the amount of activated CASP8, we can divide this phase into two types. In the type of stage when the amount of active CASP8 is higher, direct activation of CASP3 occurs. However, in the second type, when we are dealing with a low level of CASP3 activation via CASP8, caspase-8 activates the pro-apoptotic Bid protein into the active form tBid, which interacts with Bax/Bak proteins, and CytC is released [84,89,90].

The second group of death receptors includes the TNFR1 (DR1), TRAMP (DR3), DR6, and EDAR receptors. The apoptosis-inducing ligand is TNF. Once activated, the receptors recruit the TNF-related death domain (TRADD) as an adapter protein and bind to TNF receptor-related factors-2.5 (TRAF2.5), receptor-interacting protein kinase (RIP1 or RIPK1), and cellular inhibitors of apoptosis proteins (cIAP) [84].

Two distinct complexes may be formed for TNFR1. Complex I is the TNFR1 complex consisting of proteins containing DD TRADD, TNF-2 receptor-associated factor (TRAF2 (TNF)), cIAP1, and the receptor serine/threonine protein kinase 1 (RIP1). This complex activates nuclear factor-κB (NF-κB) and the c-Jun NH2-terminalkinase/Jun proto-oncogene/AP-1 transcription factor subunit (JNK/AP-1). NF-κB inhibits TNF-induced apoptosis. Complex II arises after the TRADD-based complex cleaves from the receptor and recruits FADD and the initiator CASP8. The balance between these complexes is dependent on the protein FLIP, a CASP8 inhibitor. Complex II mediates apoptosis when activation of the NF-κB pathway is inadequate [84].

In the classic way of NF-κB activation, after the action of an appropriate stimulating factor, e.g., pro-inflammatory cytokines (TNF), the IKK kinase complex is activated. The active IKK complex catalyzes the phosphorylation of IκBα, which leads to its degradation and the release of the NF-κB dimer. Then, the NF-κB dimer is translocated to the cell nucleus and activates the transcription of appropriate genes, the end products of which have the ability to inhibit apoptosis [91,92,93].

In addition to DR, apoptosis is influenced by growth factors through the phosphatidinositide-3-kinase (PI3K) and murine thymus virus oncogene homolog v-AKT (AKT) pathways. Growth factors bind to receptors and activate PI3K, which activates AKT. AKT is important in the regulation of Bad. Moreover, protein kinase-C (PKC) influences apoptosis by activating ribosomal S6 kinase (p90RSK) [94].

In healthy breast cells, a balance is maintained between cell proliferation and death. Once a balance is disrupted, an activated anti-apoptotic signaling pathway or a deficiency in the pro-apoptosis pathway can lead to uncontrolled cell proliferation [95,96].

Withaferin A and extracts from the Ayurvedic medicinal plant Withania somnifera can inhibit the proliferation of breast cancer cells by inducing apoptosis in both the intrinsic and extrinsic pathways. WA and/or WS root protein extract (WSPF) induces death in cultured MDA-MB-231 and MCF-7 cancer cells and MDA-MB-231 xenografts in vivo by mediating the production of reactive oxygen species (ROS) in the intrinsic apoptosis pathway (Figure 2) [28,97,98]. Selective killing of MCF-7 and MDA-MB-231 cancer cells by WA and/or WSPF is mediated by the induction of oxidative stress via ROS levels, DNA damage, mitochondrial structure, and membrane potential [97,98,99]. WA inhibits oxidative phosphorylation (OXPHOS) in Complex III, accompanied by apoptotic release of DNA fragments associated with histones in the cytosol [28,100,101]. However, the anti-cancer effect of WA was significantly attenuated in the presence of anti-oxidants, as it has been shown that ectopic expression of Cu and Zn-superoxide dismutase (SOD) significantly weakens its apoptotic properties [28]. Moreover, WA shows high selectivity, causing ROS production only in MDA-MB-231 and MCF-7 cells, but not in the normal human mammary epithelial cell line (HMEC) [28].

WA is involved in the regulation of the expression of Bcl-2 proteins, which are critical regulators of cell death, acting either as inhibitors or facilitators of apoptosis. Both Bax and Bak appear to contribute to the induction of apoptosis by WA (Figure 2). The active substance contained in Ashwagandha enhances the activation of these pro-apoptotic proteins in MDA-MB-231 and MCF-7 cells [28]. WA inhibits the survival of MCF-7 and MDA-MB-231 breast cancer cell lines in culture and delays the growth of MDA-MB-231 xenografts in vivo due to reduced cell proliferation and increased apoptosis. According to Stan et al., apoptosis by WA is probably mediated by forkhead box O3 (FOXO3a) and Bim proteins in both cancer cell lines. WA-induced apoptosis was accompanied by the induction of Bim-s and Bim-L in MCF-7 cells and the induction of Bim-s and Bim-EL isoforms in MDA-MB-231 cells [25].

WA also results in suppression of these IAP family proteins: XIAP, cIAP-2, and survivin in MDA-MB-231 and MCF-7 human breast cancer cells (Figure 2). IAP family proteins (IAP, cIAP1, cIAP2, NAIP, livin, and survivin) act as endogenous inhibitors of apoptosis and are involved in the cell’s adaptive response to stress, differentiation, motility, and immune response. IAPs can inhibit both the intrinsic and extrinsic apoptosis pathways. The overexpression of IAP family proteins is observed in breast cancer, which results in improved survival of cancer cells, increased tumor growth and, consequently, metastases. Therefore, it seems that the reduction in the expression of these proteins by WA indicates its significant role in apoptosis [102].

In the extrinsic apoptosis pathway, the TRAIL pathway is considered the most attractive cancer therapy agent, which is non-toxic compared to the FasL and TNF-α pathways. Its safety in cancer patients has been confirmed in phase I and II clinical trials [103,104,105,106]. TRAIL is expressed mainly on the surface of immune cells and induces apoptosis in various cancer cell lines. In a group of patients with triple-negative breast cancer (TNBC), pre-clinical models have demonstrated the effectiveness of therapy targeting the TRAIL DR pathway [107]. WA inhibits breast tumor growth by upregulation of death receptor 5 (DR5, also known as TRAIL-R2/TRICK-2/KILLER/TNFRSF10B) (Figure 2) [107]. WA has been shown to activate phosphorylation of S6RSK in breast cancer cells via activation of cell signal-regulated kinase (ERK) [108]. Both RSK and ERK play a key role in breast cancer progression and metastasis [109]. WA induced a feed-forward loop of ERK and RSK, causing the simultaneous upregulation/activation of homology protein/C-EBP axis (CHOP) and ETS-like transcription factor 1 (Elk1). The recruitment of CHOP and Elk1 to the DR5 promoter leads to activation of the death receptor, DR5, which is responsible for the aggregation of proteins promoting apoptotic signal transduction [108].

The heat shock protein (HSP) transcription factor may also play an important role in apoptosis through WA by regulating survivin expression. In many types of cancer, heat shock factor 1 (HSF1) is a transcription factor activated under environmental stress, which leads to increased expression of HSP proteins. HSF1 is increased in expression and facilitates cancer transformation by modulating signaling pathways related to growth and proliferation, apoptosis, metabolism, and motility. WA induced HSF1 phosphorylation, which is probably due to a transient cellular defense response to WA-induced stress [110]. The level of HSP expression determines the fate of cells, because these biomolecules can direct them to the apoptosis or survival pathway. One of the HSP subfamily proteins, heat shock chaperone 90 (HSP90), has become an exciting target for cancer therapy due to its role in regulating cell proliferation, survival, and apoptosis. HSP90 can modulate the activity and stability of many transcription factors and kinases related to apoptosis, including NF-κB, p53, protein kinase B (PKB/AKT), Proto-Oncogene, Serine/Threonine Kinase (Raf-1), and stress-activated protein/kinase/Jun N-terminal kinase (SAPK/JAK). In the pathway involving NF-κB, cell survival is conditioned by the formation of an NF-κB complex with the kinase of the κB inhibitor kinase complex (IKK). KK is composed of IKKα and IKKβ and NF-κB essential modulator (NEMO) or IKKγ. IKKβ IKK kinase phosphorylates two specific serine residues in the N-terminal region of IκBα, causing proteasomal degradation of IκBα and nuclear translocation of canonical NF-κB members, mainly p50/RelA and p50/c-Rel dimers [111,112]. The active substances of Ashwagandha, 2,3-unsaturated double bond-containing withanolides, exhibit potent inhibitory effects on the activity of the Hsp90 chaperone by depleting several important signaling molecules involved in cell survival (Figure 2). Withanolides inhibited the IKK/NF-κB pathway in MDA-MB-23, an ER-negative human breast cancer cell line. They reduced the activity of anti-apoptotic proteins (Bcl-2, Bcl-xL, and c-FLIP) that are regulated by NF-κB [113].

Janus kinase (JAK)-signal transducer and activator of transcription (STAT) signaling also mediates apoptosis. STAT proteins (STAT 1A, 1B, 2, 3, 4, 5A, 5B, and 6) are a family of transcription factors, each of which performs a unique function in the transmission of extracellular and direct signals regulating the transcription of genes that are involved in cell survival, proliferation, chemoresistance, and angiogenesis. Signal transducers and activators of transcription proteins (STATs) are activated by binding to their receptors on the cell membrane. The phosphorylated receptor-kinase complex is the site of STAT attachment, which is then phosphorylated on tyrosine residues necessary for activation. Then, after dimerization, STAT translocated to the nucleus can influence cell apoptosis by regulating the transcription of target genes such as Bcl-xL, p21, and Myc [114,115,116].

**Figure 3:**
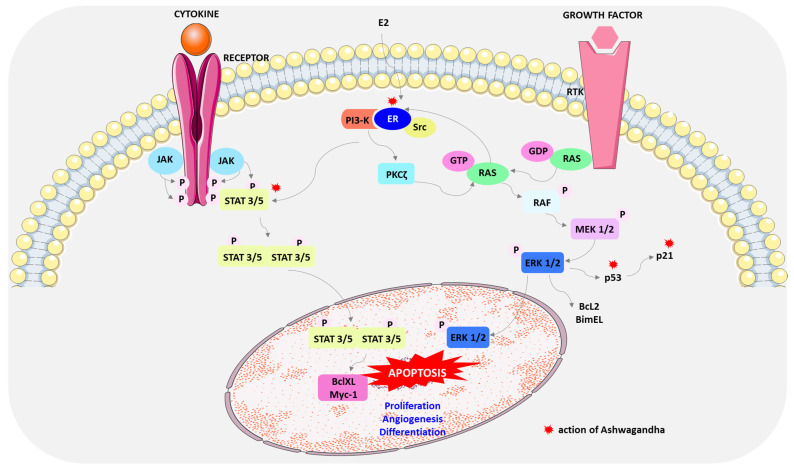
Mechanism of action of Ashwagandha. The effects of Ashwagandha on pathways of apoptosis is marked with a red asterisk. Active components of Ashwagandha induce apoptosis by inhibiting the recruitment of STAT3 and STAT5, regulating the MAPK pathway and the expression of p53 and p21 proteins and reducing the level of estrogen receptor alpha (Erα). Abbreviations: AKT—protein kinase B; Bcl-XL, BimEL, Myc-1—Bcl-2 family proteins; E2—17β-estradiol; ER—estrogen receptor; ERK—extracellular signal-regulated kinase; FOXO3a—forkhead transcription factor; GDP—guanosine diphosphate; GTP—guanosine-5′-triphosphate; JAK—janus kinase; MEK—mitogen-activated extracellular signal-regulated kinase; NF-κB—nuclear factor-κB; PI3-K—PI 3-kinase; p21 protein—Cyclin-dependent kinase inhibitor; p53 protein—transcription factor with tumor suppressor properties; RAF—rapidly accelerated fibrosarcoma kinase; RAS—cellular signal transduction protein; RTK—receptor tyrosine kinase; STAT—signal transducer and activator of transcription. This figure was created using Servier Medical Art (available at https://smart.servier.com/, accessed on 12 April 2024).

In many human cancers, including breast cancer, STAT3 and STAT5 are persistently phosphorylated and overactivated [117]. STAT3 increases the expression of c-Myc and the metastasis regulator Twist genes and is, therefore, thought to induce breast cancer [118]. Aberrant STAT5 signaling promotes cyclin D, Bcl-2, and matrix metalloproteinase-2 (MMP-2) gene expression, resulting in increased cell proliferation, survival, and metastasis [119,120]. WA causes inactivation of STAT3 and STAT5 by inhibiting their recruitment to growth factor and cytokine receptors as well as tyrosine phosphorylation, nuclear translocation, and DNA binding. Furthermore, STAT3 inhibits Bcl-xL and Mcl-1. (Figure 3) [117].

Other studies have demonstrated the role of mitogen-activated protein kinases (MAPKs) in the regulation of apoptosis by WA (Figure 3). MCF-7 cell death resulted from the action of WA via pharmacological inhibition of ERK kinase and p38 MAPK [121]. The MAPK pathway, often called the RAS-RAF-MEK-ERK signaling cascade, serves to transmit upstream signals to downstream effectors to regulate physiological processes such as cell proliferation, differentiation, survival, and death [122]. It is the most frequently mutated signaling pathway in human cancers; therefore, this pathway may be a promising strategy in cancer therapy [123,124].

The mitogen-activated protein kinase (MAPK) cascade consists of serine/threonine kinases that convert extracellular molecules such as growth factors (EGF), insulin-like growth factor I (IGF-I), hormones, and differentiation factors into intracellular signals [123,124]. There are four core protein kinases in the Ras/Raf/MEK/ERK-signaling pathway: Ras, Raf, MEK, and ERK. Once the signaling molecules are attached, the receptors dimerize, which activates RAS proteins. Activated RAS protein recruits RAF protein from the cytosol to the cell membrane, leading to its activation. Active RAF kinase phosphorylates MEK1 and 2 proteins, which activate ERK1 and 2 proteins. After transmitting the signal to the cell nucleus, ERK kinases phosphorylate and activate many target molecules responsible for cell growth, migration, and survival [125,126].

WA also acts to reduce the level of estrogen receptor alpha (Erα), which consequently affects the induction of apoptosis and inhibition of the growth of ER-α-positive MCF-7 and T47D breast cancer cells [33].

17β-estradiol (E2) exerts its effects on proliferation mainly through rapid non-genomic mechanisms derived from the binding of the hormone to Erα. Non-genomic signals are transmitted to the nucleus via various intracellular-signaling pathways such as MAPK/ERK and Pl3K/AKT [127,128,129]. E2 induces the association of ERα with Src and the PI 3-kinase (PI3K) adapter subunit p85a. Estradiol-stimulated PI 3-kinase/PDK1 pathway activates PKCζ in MCF-7 cells, which controls Ras-dependent ERK activation. Furthermore, E2 phosphorylates STAT3 and STAT5 by an ER-dependent mechanism [130]. The ERβ isoform has also been shown to play an important role in the proliferative action of E2, which can act as a tumor suppressor by modulating the proliferative action of ERα [129]. The E2-ERβ complex activates p38/MAPK leading to apoptosis while E2-ERα activates transduction pathways involved in cell cycle progression. The E2-ERβ complex activates p38/MAPK leading to apoptosis while E2-ERα activates transduction pathways involved in cell cycle progression [129,131,132]. WA reduces ERα levels post-translationally, inducing ERα protein aggregation and degradation. The anti-cancer activity of WA is mediated by RET tyrosine kinase (RET) and p53, which inhibits growth and apoptosis [110,133]. While RET is overexpressed in ERα-positive breast cancer, its activation stimulates the proliferation, survival, and dispersal of MCF-7 breast cancer cells [134]. WA had anti-tumor effects through the downregulation of RET protein with parallel depletion of Erα coupled with increases in the expression of phosphorylated mitogen-activated protein kinase p38 (phospho-p38 MAPK), p53, and p21 (Figure 3) [14,110].

In addition to apoptosis, there are also other non-apoptotic modes of cell death including autophagy, necroptosis, paraptosis, and apoptosis-like programmed cell death.

Autophagy or autophagic cell death are referred to as type II cell death. This process plays an important role in the degradation of cellular components inside the dying cell in autophagic vacuoles. It helps maintain cellular energy supply and homeostasis in rapidly proliferating cancer cells. Cancer cells use this process to respond to various environmental stimuli and avoid anti-cancer therapy [135]. The anti-tumor effect of WA in many breast cancer subtypes, including luminal A, luminal B, basal, claudin-low, and HER2W subtypes, is related to impairment of lysosomal activity, causing blockage of autophagic flux, which results in energy depletion leading to growth inhibition and induction of apoptosis. WA is an activator of 5′AMP-activated protein kinase AMP (AMPK), which works synergistically with 2-deoxy-d-glucose (2-DG) to inhibit breast cancer growth and is also an inhibitor of lactate dehydrogenase (LDHA), a key enzyme catalyzing the conversion of pyruvate to lactate [136].

Inhibition of the WA-mediated proteasome degradation system and perturbation of autophagy causes the accumulation of ubiquitinated proteins, which in turn results in unfolded protein responses and ER stress-mediated proteotoxicity in the human breast cancer cell lines MCF-7 and MDA-MB-231 [137].

Another process involved in programmed cell death that is morphologically different from apoptosis and autophagy is paraptosis. WA acts on the mitochondrial membrane potential, causing its hyperpolarization and the formation of many cytoplasmic vesicles. In the human breast cancer cell lines MCF-7 and MDA-MB-231, mitochondrial swelling and fusion occur, and the endoplasmic reticulum (ER) expands. WA reduces the level of the native paraptosis inhibitor, actin-interacting protein-1 (Alix/AIP-1), indicating that WA promotes death in both MCF-7 and MDA-MB-231 cell lines through paraptosis through the action of ROS [138].

Summarizing pre-clinical studies, it can be concluded that Ashwagandha has anti-cancer activity in various experimental models. WS acts at many stages, leading to apoptosis, both in the extrinsic and intrinsic pathways. WA and/or its extracts induce death in cancer cell cultures in vivo by mediating ROS production [28,97,98,99]. WA regulates the expression of Bcl-2 proteins, reduces the expression of IAP proteins XIAP, cIAP-2, and survivin and apoptosis inhibitors [25,97,102]. It inhibits the growth of breast cancer cells by activating DR5 [108]. It affects the activity of the Hsp90 protein, the recruitment of STAT3 and STAT5, and the regulation of the MAPK pathway [107,117,121]. It influences the reduction of the level of estrogen receptor alpha [33,110]. WA inhibits autophagic flux and reduces the level of paraptosis inhibitor, inducing death in breast cancer cells via ROS [137,138].

Information regarding the mechanism of action of Ashwagandha on various cell lines, taking into account the concentrations of active components of the plant extract used, is presented in Table 1.

There is little information about clinical trials confirming the use of WS in the treatment of breast cancer, although they are crucial to confirm its effectiveness and safety. It has only been shown that WS reduces chemotherapy-induced fatigue and improves the quality of life after administering 2 g of WS root extract every 8 h, throughout the course of chemotherapy. Similarly, survival analysis showed that patients in the WS treatment group had a better 24-month survival rate of 76% compared to the control group, which had a survival rate of 56% [47,139,140].

Although Ashwagandha was considered safe in clinical trials, improper dosage or failure to take into account individual contraindications may lead to a number of health problems, ranging from nausea and vomiting to more serious complications, such as hypertension, liver dysfunction, and hyperbilirubinemia [141,142].

Additionally, people taking anti-diabetic, anti-hypertensive, or central nervous system medications should be careful, because WS may interact with these medications, enhancing or changing their effects. Ashwagandha may also affect thyroid hormone and testosterone levels [143,144,145,146].

There is also a lack of clear evidence on the safety of the long-term use of WS over many months or years. Ashwagandha was well tolerated at a dose of 300 mg per day in an eight-week study [147]. For insomnia, a dose of 600 milligrams per day has been found to be safe and effective [148]. This dosage has also been associated with improved memory [149].

The extensive exploratory studies demonstrate the potential of Ashwagandha in the treatment of breast cancer through its anti-cancer activity, safety profile, and combination therapy possibilities. However, due to inconsistent therapeutic results resulting from the widely varying composition of active components in the plant extract, the actual use of WS in the treatment of cancer is limited. Further research and standardization are needed to harness its full therapeutic potential.

## 5. Conclusions

Withaferin A (WA) and withanolides are the most promising anti-cancer compounds of Ashwagandha, which play a major role in the induction of the apoptosis of cancer cells. This review attempts to confirm its therapeutic properties, with a particular emphasis on its role in the treatment of breast cancer. The literature data indicate that compounds isolated from various parts of this plant, such as the root, stem, and leaves, have significant anti-cancer and immunomodulatory properties; therefore, they can be used alone or in combination with other chemotherapy drugs in the treatment of breast cancer. Reaching for natural medicine can complement the standard treatment of cancer patients without undesirable side effects and may also have a positive impact on reducing the feeling of fatigue and improving their quality of life [139].

## Figures and Tables

**Table 1:** Effect of WA and Ashwagandha extract on various breast cancer cell lines.

Phytochemicals/ Concentration	Cell Lines	Mechanism of Action of Ashwagandha	Reference
WA/2.5 μM	MDA-MB-23 MCF-7 MDA-MB-231	ROS-mediated apoptotic induction due to inhibition of mitochondrial respiration.	[28]
WA/3.0 mg/mL	MCF-7 MDA-MB-231	ROS-mediated apoptotic induction. Dysregulation of Bax/Bcl-2, loss of mitochondrial membrane potential and caspase-3 activation.	[97]
Extract/6 μg/mL; WA/1 μM, witanonem/25 μg/mL	MCF-7	ROS-mediated apoptotic induction. DNA damage, mitochondrial structure, and membrane potential.	[98]
WA/2 μM	MCF-7 i SUM159	Changes the assembly of complex III. Inhibition of mitochondrial dynamics.	[99]
WA/IC50 < 2 μM	MCF-7 MDA-MB-231	Regulation of apoptosis involving FOXO3a and Bim. Induction of Bim-s and Bim-L in MCF-7 cells. Induction of Bim-s and Bim-EL isoforms in MDA-MB-231 cells.	[25]
WA/2.5 and 5 μM	MDA-MB-231 MCF-7	Decrease in the expression of XIAP, cIAP-2 and survivin proteins, apoptosis inhibitors.	[102]
WFA/5 μM	MCF7 MDA-MB-231	DR5 upregulation. Increased nuclear accumulation of Elk1 and CHOP.	[108]
WA/5 μM, 4β-Hydroxywithanolide (HW)/20 μM, Anomanolide A (AA)/20 μM, Peruvianolide H (PH)/20 μM)	MDA-MB-231 MCF-7	Inhibition of Hsp90.	[107]
WA/3 μM	MDA-MB-468	Inhibition of STAT3 and STAT5 recruitment.	[117]
WA/2.5 μM	MCF-7	Inhibition of ERK and p38 MAPK.	[121]
	MCF-7 T47D	Inhibition of estrogen receptor α expression.	[33]
WA/2.5, 5 μM	MCF-7		[110]
WA/5 μM	MCF7, MDA-MB-231, MDA-MB-468, T47D, SUM149, SUM159, SKBR3	Blocking the flow of autophagy and lysosomal proteolytic activity.	[136]
WA/4 µM	MCF-7 MDA-MB-231	Inhibition of the proteasome degradation system and disruption of autophagy.	[137]
	MCF-7 MDA-MB-231	ROS-mediated paraptosis induction.	[138]
